# Sounds Reset Rhythms of Visual Cortex and Corresponding Human Visual Perception

**DOI:** 10.1016/j.cub.2012.03.025

**Published:** 2012-05-08

**Authors:** Vincenzo Romei, Joachim Gross, Gregor Thut

**Affiliations:** 1 Institute of Neuroscience and Psychology, University of Glasgow, 58 Hillhead Street, Glasgow G12 8QB, UK; 2 Wellcome Trust Centre for Neuroimaging at UCL, Institute of Neurology, 12 Queen Square, London WC1N 3BG, UK; 3 UCL Institute of Cognitive Neuroscience, 17 Queen Square, London WC1N 3AR, UK

## Abstract

An event in one sensory modality can phase reset brain oscillations concerning another modality [1–5]. In principle, this may result in stimulus-locked periodicity in behavioral performance [6]. Here we considered this possible cross-modal impact of a sound for one of the best-characterized rhythms arising from the visual system, namely occipital alpha-oscillations (8–14 Hz) [7–9]. We presented brief sounds and concurrently recorded electroencephalography (EEG) and/or probed visual cortex excitability (phosphene perception) through occipital transcranial magnetic stimulation (TMS). In a first, TMS-only experiment, phosphene perception rate against time postsound showed a periodic pattern cycling at ∼10 Hz phase-aligned to the sound. In a second, combined TMS-EEG experiment, TMS-trials reproduced the cyclical phosphene pattern and revealed a ∼10 Hz pattern also for EEG-derived measures of occipital cortex reactivity to the TMS pulses. Crucially, EEG-data from intermingled trials without TMS established cross-modal phase-locking of occipitoparietal alpha oscillations. These independently recorded variables, i.e., occipital cortex excitability and reactivity and EEG phase dynamics, were significantly correlated. This shows that cross-modal phase locking of oscillatory visual cortex activity can arise in the human brain to affect perceptual and EEG measures of visual processing in a cyclical manner, consistent with occipital alpha oscillations underlying a rapid cycling of neural excitability in visual areas.

## Highlights

► Sound modulates visual perception by phase-locking occipital alpha phase ► Visual perception cocycles with the phase alignment of occipital alpha oscillations ► Alpha rhythm causally shapes perception in a periodic fashion ► Occipital alpha phase indexes excitability of visual areas

## Results

**Figure 1:**
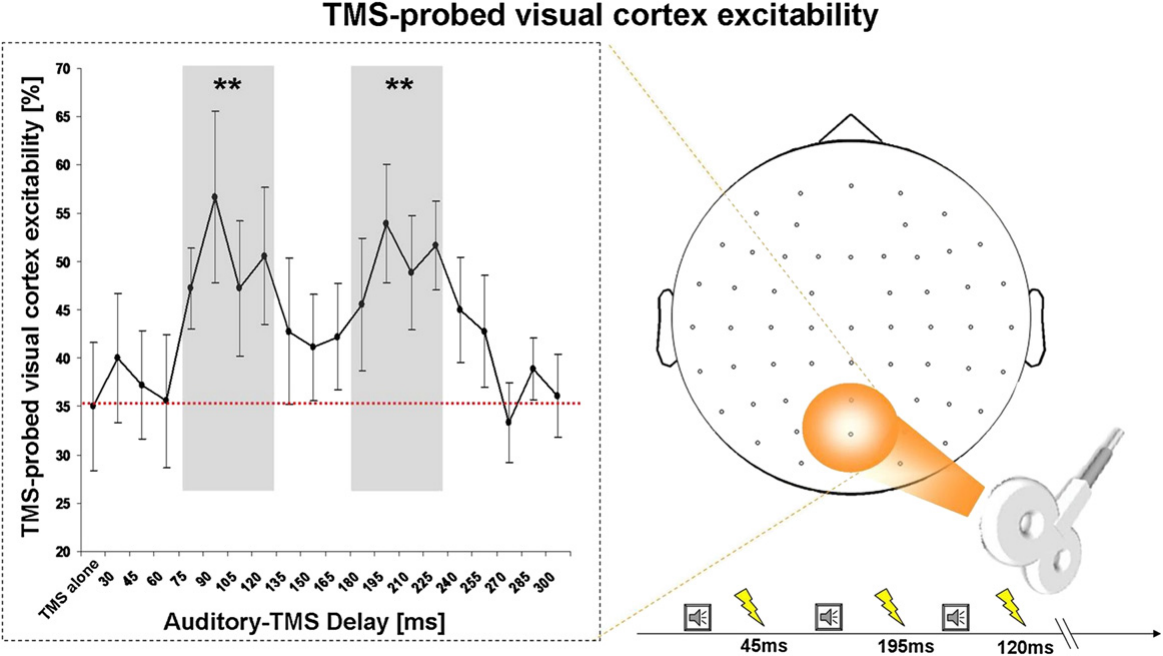
Paradigm and Results of Experiment 1 Right panel shows schematic of the paradigm, illustrating TMS over occipital cortex in blindfolded participants (plus concurrent EEG recordings for experiment 2). TMS was applied at 85% of phosphene threshold, after different delays following a salient sound, or with no sound in baseline condition. Left panel shows percentage of trials with TMS-induced phosphene reported (±SEM), against delay since sound onset, for experiment 1. Leftmost point is no-sound baseline (BSL). The shaded areas (75–120 ms and 180–225 ms) represent windows of significantly increased visual cortex excitability by auditory input, i.e., phosphene rate > BSL, ^∗∗^p < 0.01 Bonferroni corrected. Note the periodicity of phosphene perception over time, which cycles at around 10Hz (∼100 ms peak-to-peak).

We hypothesized that a sound may phase-align oscillatory alpha activity over occipital areas (typically around 8–14 Hz) and thereby reveal cyclical influences of visual brain rhythms on subsequent visual processing. We tested this by assessing in two experiments reports of phosphene perception (using occipital transcranial magnetic stimulation [TMS] as in [10, 11]) at various time points after a critical sound (Figure 1, right panel) and by also measuring electroencephalography (EEG) in the second experiment.

### Experiment 1: Phosphene Perception Shows ∼10 Hz Periodicity Time-Locked to the Presentation of a Short Sound

The rate of phosphene perception for occipital TMS provides an established measure of excitability for visual cortex. On each trial of experiment 1, a single TMS pulse was delivered at one of 19 time points between 30 and 300 ms postsound or without a sound in the baseline condition. The results revealed a cyclical pattern of phosphene perception rate, time-locked to the critical sound (Figure 1, left panel). Percentage of induced phosphenes differed significantly in relation to sound onset (F(19,152) = 2.16; p = 0.005) with maximal values (highest rate of phosphene perception) occurring in two time windows (Figure 1, left panel). The first peak in phosphene perception rate arose between 75 and 120 ms (reproducing [10]) and the second peak between 180 and 225 ms (at previously unexplored delays). Comparing the TMS-only baseline (no preceding critical sound, leftmost point in Figure 1, left panel) with performance in windows centered on the apparent “alpha”-like peaks and troughs confirmed enhanced phosphene perception in 75–120 ms and 180–225 ms windows (Bonferroni corrected paired t tests: all p < 0.01). Phosphene perception in 30–60 ms, 135–165 ms, and 255–300 ms windows did not differ significantly from baseline, i.e., the no-sound condition (all p > 0.33).

Thus, phosphene perception followed a cyclical pattern (best-fitting sine wave: 9.6 Hz, Rsquare: 82% of variance explained), with roughly ∼100 ms peak-to-peak interval, consistent with the ∼10 Hz nature of occipitoparietal alpha activity, previously linked to visual perception [12–21]. Because the phosphene data in Figure 1 (left panel) are all time locked to the critical sound, this suggests a possible phase locking of the perceptually relevant, occipitoparietal alpha activity by the critical sound.

### Experiment 2: Phosphene Perception Correlates with Phase Dynamics of Occipital Alpha Activity after Phase Locking to the Sound

**Figure 2:**
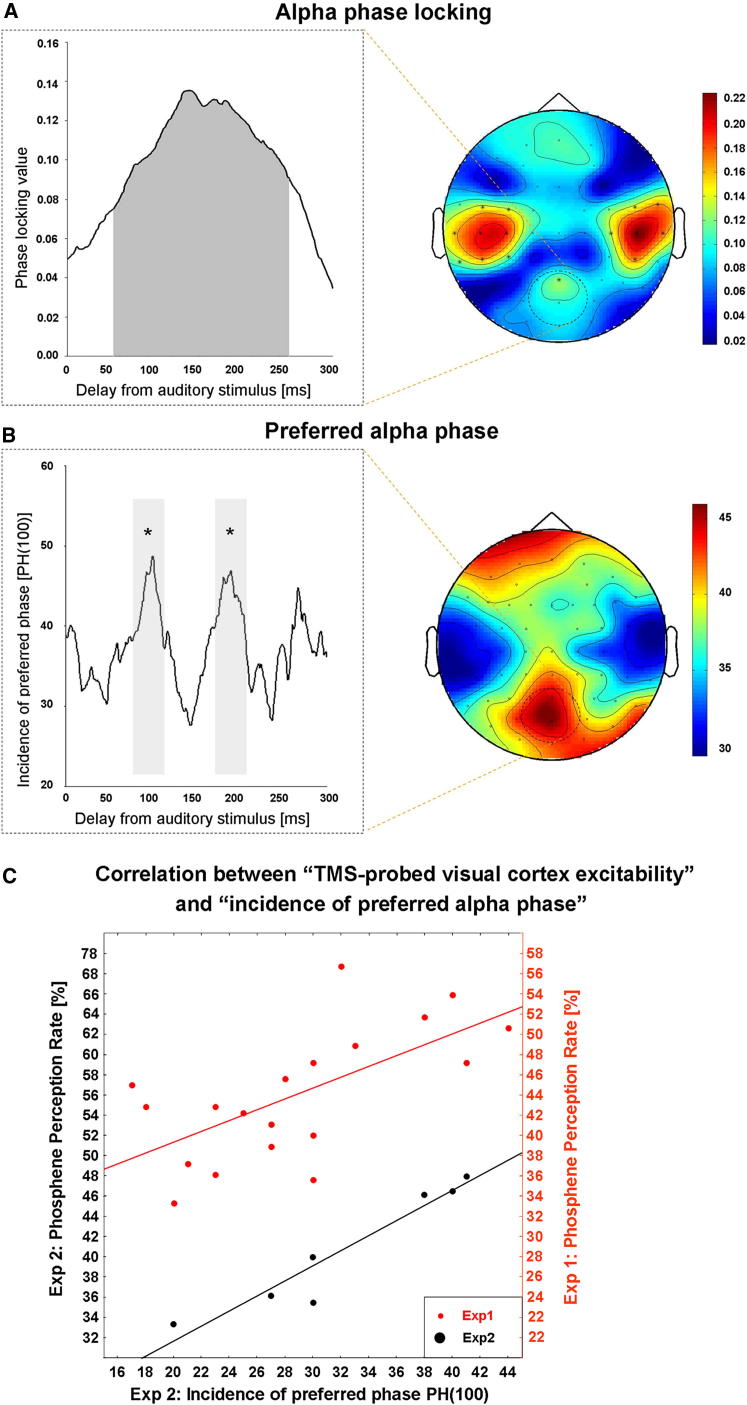
Alpha Phase Locking for No-TMS Trials in Experiment 2 (A) Instantaneous EEG phase, time locked to sound onset, was computed with the Hilbert transform after applying a band-pass filter (8–14 Hz). Right panel shows the 10 Hz phase-locking topography, with the effect evident in sensors overlaying not only auditory but also highlighted parieto-occipital cortex. Left panel depicts 10 Hz phase-locking for highlighted parieto-occipital sensors, which was significant (p < 0.001) between 50 ms and 250 ms following auditory stimulus onset (shaded window). (B) Preferred alpha phase at 100 ms delay. Circular statistics were used to estimate PH(100), the mean instantaneous phase at 100 ms post sound (the time of highest sound-induced enhancement of phosphene detection rate in experiment 1). The number of trials within ±10 degrees of PH(100) is shown at left against time since sound, with corresponding topography at right. Note that the fluctuation in number of trials showing preferred phase PH(100) peaks not only at 100 ms (by definition), but also at around ∼200 ms, with the resulting cyclical pattern closely resembling the periodicity of perceived phosphene rate shown in Figure 1, in terms of peak-to-peak interval and peak latencies (i.e., frequency and phase). The shaded windows highlight that PH(100) not only at 100 ms (±20 m) but also at 200 ms (±20) was significantly higher than baseline (BSL) or the 150 delay (±20ms), all ^∗^p < 0.05 Bonferroni corrected. (C) EEG-phosphene correlation for separate datasets from experiments 1 and 2 (same participants). Scatterplot with red points and red line shows that the number of no-TMS EEG trials showing a phase within PH(100) at 19 equally spaced time points in the window 30 ms to 300 ms (experiment 2) correlates with the corresponding phosphene rates (scored along right axis) from separate experiment 1 (Figure 1). Please note the baseline (no-sound) condition was not considered in this analysis, as a time window relative to sound onset cannot be set for the preferred-phase EEG measure without a sound. Scatterplot with black points and black line shows a similar correlation between phosphene perception rate on the TMS trials in experiment 2 (scored along left axis) and dynamics of preferred phase for no-TMS trials in the same experiment.

In experiment 2, we acquired concurrent EEG in the same paradigm and the same participants as in experiment 1, but with fewer TMS time points (now 7 rather than 19, see Experimental Procedures) to allow more trials in each condition for EEG analysis and a higher proportion (1/8) of no-TMS trials. Phosphene perception rates were highly correlated between experiments 1 and 2 (r(7) = 0.90; p < 0.003), for the sound-TMS relations that were in common between these two studies, i.e., experiment 2 essentially reproduced the ∼10 Hz cyclical pattern of phosphene perception of experiment 1 (see below for more detailed analysis of this pattern). We next analyzed EEG data from experiment 2, initially for sound-only trials (no TMS) because these can provide a pure measure of phase locking of EEG activity to the critical sound in the absence of TMS (thus without any associated visual percept). Significant 10 Hz phase locking (p < 0.001) to the critical sound was evident not only for sensors overlaying auditory cortex but also over parieto-occipital cortex, from 50 ms to 250 ms following auditory stimulus onset (Figure 2A). Note that none of the frontal electrodes showed significant phase locking. We then calculated the number of trials showing a phase for parieto-occipital electrodes within ±10 degrees of the preferred phase at 100 ms (the time point postsound at which phosphene rate peaked in experiment 1). This measure showed a cyclical pattern, being significantly higher not only around 100 ms (by definition) but also around 200 ms after the sound, as compared to the 150 ms delay or the no-sound baseline (all p < 0.05, Bonferroni corrected); see Figure 2B. This pattern is strikingly reminiscent of the phosphene rate data from the previous experiment (Figure 1) and the correlated phosphene perception rate of the new experiment.

For another view on the same data and to better illustrate the relationship of EEG phase with phosphene perception, we plotted the alpha-phase dynamics over time since sound onset against the phosphene rate over time (Figure 2C) and calculated correlation coefficients. The red dots and line in Figure 2C plot phosphene perception rate for different time points in experiment 1 (taken from Figure 1, left panel) against the preferred phase [PH(100)] EEG data for the same time points in experiment 2 (from Figure 2B). The black dots and line in Figure 2C plot the corresponding phosphene rate data from experiment 2, against the same preferred phase EEG data. Alpha-phase dynamics (EEG data on no-TMS trials from experiment 2) were significantly correlated with the corresponding phosphene rate in the separate experiment 1 (r(18) = 0.65 p < 0.003, red dots and line); and also with the phosphene rate for TMS trials in the same experiment 2 (r(6) = 0.96; p < 0.0008, black dots and line).

Control analysis showed that the relationship between sound-locked occipitoparietal activity and cyclic phosphene perception rate is limited to the alpha band, because there was no comparable effect in other frequency bands above and below alpha activity (for further results and discussion see Supplemental Information: Control Analysis in Nonalpha Flanker Frequency Bands and Figure S1). This therefore supports phase locking of posterior alpha activity as the underlying cause of the periodicity of perception.

### Individual Phosphene Perception Frequency Correlates with Individual Alpha Frequency

To further examine the link between periodicity in phosphene perception and cyclic brain activity over posterior areas, we related the periodicity of phosphene perception to the frequency of ongoing EEG alpha activity that is prominently observed at baseline (prior to sound onset). We obtained a significant correlation between the frequency of best-fitting sinusoids in individual phosphene perception rates and the individual alpha frequency (IAF), the latter estimated from presound EEG recordings (r = 0.67; p = 0.024, see Supplemental Information: Correlation between Periodicity in Phosphene Perception and Individual Alpha Frequency and Figure S2). This therefore provides a further piece of evidence that the sound-locked cyclic behavior in phosphene perception is linked to alpha oscillations.

### Phosphene Perception Correlates with EEG-Probed Visual Cortex Reactivity

**Figure 3:**
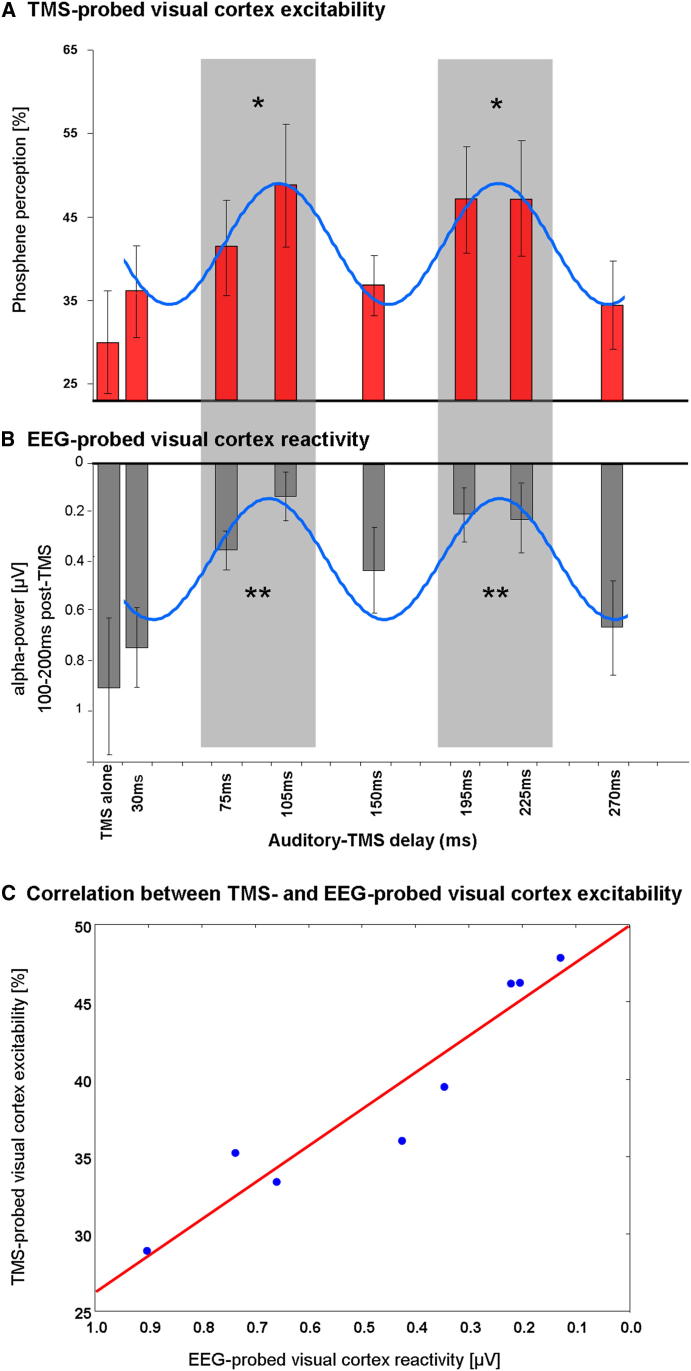
Results for TMS-EEG Trials in Experiment 2 (A) TMS-probed visual cortex excitability as indexed by rate of phosphene perception, for the eight conditions (±SEM) relative to a preceding sound (baseline with no sound, plus seven delays after sound). Note the cyclical pattern, with significantly more phosphene reports arising within the two shaded time windows (significantly greater than baseline = no sound, ^∗^p < 0.05 Bonferroni corrected t tests). (B) EEG-probed visual cortex reactivity to the TMS pulses (±SEM). Visual cortex reactivity is estimated by alpha-power changes in a post-TMS time window (TMS locked), as a function of delay relative to critical sound. High reactivity is indicated by low alpha values and low reactivity by high alpha values (y axis; note the inverse scaling with low alpha values plotted upwards and high values downwards). The shaded areas (75–105 ms and 195–225 ms delays after sound) represent windows of significantly enhanced visual cortex reactivity (reduced parieto-occipital alpha activity) after a TMS pulse (i.e., reactivity significantly above baseline, ^∗∗^p < 0.015 Bonferroni corrected t tests). Note the periodicity in visual cortex reactivity that is once again found to cycle at around ∼10Hz. (C) Scatterplot showing the close relation between rate of phosphene perception and visual cortex reactivity for each TMS condition in experiment 2.

Finally, we turn to the EEG-TMS trials from experiment 2. Rate of phosphene perception for occipital TMS at the seven time points after the sound (plus no-sound baseline) is shown in Figure 3A, illustrating its cyclical pattern (blue sinusoid represents best fitting sine wave; 9.1 Hz, Rsquare: 96% of variance explained). Phosphene rate varied in relation to the sound (F(7,56) = 2.77; p = 0.015), being higher at 75–105 ms delays, then again at 195–225 ms, as compared to TMS without any preceding sound (all p < 0.05 on Bonferroni corrected t test). This reproduces the cyclical phosphene finding from experiment 1. Using EEG data on the same trials, we then estimated visual cortex reactivity to the TMS pulse as a function of delay from sound onset (and thus analogous to the phosphene rate analysis). To this end, we calculated for each condition event-related alpha power changes over occipitoparietal sensors, a measure of visual induced activity (cf. [22].), in a time window for which phosphene perception-related activity has previously been reported ([23]; i.e., 100–200 ms post-TMS), relative to presound baseline (see Experimental Procedures). Note that alpha power is considered to be inversely related to visual cortex activity (smaller values indicate higher reactivity; e.g., [22]) and hence might also show a cyclical pattern that could relate to that found for phosphene perception. As Figure 3B shows, this pattern was indeed confirmed. There was a significant cyclical pattern in relation to sound onset also for EEG-probed visual cortex reactivity (F(7,56) = 5.91; p < 0.00005, blue sinusoid represents best fitting sine wave; 8.6 Hz, Rsquare: 81% of variance explained). Visual cortex reactivity was significantly higher (corresponding to reduced alpha power) between 75–105 and 195–225 ms after the sound, as compared to the no-sound baseline, or to the 30, 150, or 270 ms delays (all p < 0.05 on Bonferroni corrected t tests). Again, for another view on the same data, we correlated EEG-probed visual cortex reactivity with the rate of phosphene perception across conditions within the same experiment 2. Results of this correlation showed that phosphene perception increased with visual cortex reactivity (i.e., as alpha power decreased) (r(7) = −.95, p < 0.0003); see Figure 3C.

## Discussion

These results indicate that a sound can phase lock alpha oscillations in human visual cortex, with direct consequences for perception as assessed with the phosphene measure. The rate of phosphene perception in experiment 1 showed a rapidly cycling pattern against time after sound onset (Figure 1), with two peaks of increased visual cortex excitability separated by ∼100 ms. EEG data acquired in experiment 2 after the same sound, on no-TMS trials, showed alpha phase locking not only over auditory cortex but also for posterior parietal-occipital sites implicating visual cortex (Figure 2A). The number of trials showing the preferred phase at 100 ms [PH(100)] also showed a cyclical pattern against time since sound onset (Figure 2B), peaking not only at 100 ms after the sound (by definition) but also at ∼200 ms, to produce a pattern that closely resembled the separate phosphene perception data (Figure 1). This remarkable similarity was evident in the correlation between these independently recorded variables from separate trials but of the same participants (Figure 2C, red dots and line). Finally, the TMS trials during EEG also revealed a cyclical pattern for TMS-probed phosphene perception (Figure 3A) and for EEG-probed visual cortex reactivity (using a measure of TMS-locked EEG changes at occipitoparietal sensors; Figure 3B), with these two measures correlating significantly (Figure 3C). Because the relationship between EEG-phase dynamics and phosphene perception rate were confined to the alpha-frequency band, and because the frequency of ongoing alpha oscillations matched the best-fitting frequency in the phosphene data, we conclude that the periodicity in phosphene perception reflects those of intrinsic brain rhythms. Note that the observed phase locking to the sound may either result from phase reset of ongoing oscillations, or this activity being evoked by the sound. This being said, the two alternative explanations may not be unequivocally dissociable on empirical grounds (for a detailed discussion, see [24]). But crucially, the key novel aspect of our study is the consistent finding of cyclical visual phenomena in both perceptual and EEG measures after sound onset, indicating a cross-modally triggered phase locking of perceptually relevant oscillatory alpha activity over occipitoparietal areas.

Several authors have proposed that the phase of ongoing EEG activity cycling in specific frequency bands (typically between 7 and 10 Hz, so overlapping with alpha activity) may implement a periodic sampling mechanism for perception [9, 25–27], and determines visual processing in addition to alpha power [9, 12–17]. Recent EEG studies indicate a correlative relation between prestimulus alpha phase and subsequent visual perception [17–20] or visual cortical excitability [21]. Here we build on but go beyond such work to show that mere onset of a salient sound can phase-lock human visual alpha phase, realigning periodic sampling with direct consequences for human perception, as indicated by changes in phosphene perception. Because manipulating phase also modulates perception, we infer that phase causally underlies visual cortex excitability, rather than representing a by-product. By experimental design, we also provide information on causal directionality: because the interrelated phase dynamics of brain oscillations and cortical excitability were measured in separate trials, we can exclude that the periodicity of perception has caused alpha-band activity; rather, we conclude that electrophysiological activity underlies the perceptual fluctuations and not vice versa.

Our results cannot be readily explained by the sound merely serving as a warning signal to produce temporal expectancies of when TMS (and/or visual percepts) might arise. The different time intervals after sound onset were equally likely. Although participants might potentially estimate a central tendency of this temporal distribution, this would not explain the cyclic pattern, i.e., the two distinct “peaks” shown in Figures 1, 2B, and 3A and 3B, each separated by approximately ∼100 ms (i.e., at around alpha frequency). Application of TMS itself is associated with an unavoidable “click” sound, but this was equivalent across all TMS-present conditions. Moreover we observed phase locking of parieto-occipital alpha by the critical additional sound, even when no TMS was presented (Figures 2A and 2B).

Recent research of cross-modal influences on visual processing has shown that sounds can modulate visual phosphene perception [10, 11, 28], but the neural basis of this was not identified, whereas here we uncovered phase locking of posterior alpha oscillations. Our new results are consistent with pioneering work using invasive recordings in animals [1–3], which has shown that phase alignment of one modality by another, in early sensory cortex, is an important mechanism for interrelating processing between different senses. Here we establish this for phase alignment of visual alpha in the human brain, and for its impact on conscious perception, for the first time.

Our results also add to the emerging literature on development of causal interventions for manipulating brain rhythms in humans (e.g., via rhythmic bursts of TMS [16, 29–34]; via transcranial direct or alternating current stimulation [35–40]) or in animals via invasive stimulation [41]. Our new results imply that the very simple manipulation of merely presenting a salient sound can causally impact upon visual oscillations and related visual perception and can thereby serve for experimental control of brain oscillations to probe their functional roles.

## Experimental Procedures

### Participants

Nine participants (mean age: 29.33 years, range: 22–42), six women, gave written informed consent in accord with local ethics to take part in both experiments, on separate days. For further details see Supplemental Information.

### TMS

Illusory visual percepts (phosphenes) were induced by TMS over the occipital pole via a 70 mm figure-of-eight TMS coil, connected to a Magstim Rapid2 Transcranial Magnetic Stimulator (Magstim Company, Spring Gardens, UK). An appropriate TMS site and intensity for inducing phosphenes in each individual were established in separate sessions prior to the main experiments (see Supplemental Information). For experiment 1, TMS was applied at 85% of predetermined phosphene threshold (PT), which corresponded to 60.63% (±2.65) of maximal stimulator output (MSO). The occipital TMS site was on average (±SEM) 2.81 ± 0.15 cm above the inion and 0.86. ± 0.17 cm left of midline. For experiment 2, the site was identical but PT was reassessed to take into account the increased distance between coil and skull dictated by the EEG cap. The 85% PT yielded an intensity of 63.56% (±3.79) MSO. Participants were blindfolded during all test blocks. For further details see Supplemental Information.

### Experiment 1

The critical salient auditory stimulus was a 14 ms duration 900 Hz pure tone (75 dB sound pressure level [SPL] at the ear; 44,100 kHz sampling rate) presented through two loudspeakers in front of the participants (as in [10]). TMS was applied at one of several delays after sound onset (between 30 and 300 ms in steps of 15 ms, thus for a total of 19 time delays, plus no-sound baseline on 5% of trials). Participants were instructed that in most trials a brief sound would be presented around TMS pulse delivery. Although it was stressed that the sound is irrelevant to the task (reporting phosphenes), the sound nevertheless served as a warning cue for the forthcoming TMS pulse whose delivery was expected to induce phosphenes at an invariant location but at an unpredictable time point. Twenty trials were assessed per equiprobable, intermingled condition in each participant.

### Experiment 2

The protocol was identical to experiment 1, except: (1) EEG was recorded from 64 channels; (2) possible delays between sound onset and TMS were now 30, 75, 105, 150, 195, 225, or 270 ms, whereas the remaining 1/8 of trials were sound only. Ninety-six trials were assessed per equiprobable intermingled condition. For further details on both experiments, see Supplemental Information.

### EEG Apparatus

EEG was recorded with a TMS-compatible BrainAmp 32 MR Plus unit (BrainProducts GmbH, Munich, Germany) and digitized at 5,000 Hz sampling rate (band-pass filtered at 0.01–1,000 Hz) from 64 scalp electrodes. Contact impedance was kept below 10 kOhm. TMS-compatible Ag/AgCl pellet pin electrodes (Easy cap GmbH, Germany) were mounted on the cap.

### EEG Analysis in Experiment 2

For the no-TMS trials, instantaneous EEG phase time locked to onset of the auditory stimulus was computed with the Hilbert transform, after applying a band-pass filter (fourth-order butterworth filter, 8–14 Hz). Trials were pooled across participants and the distribution of phase across trials was tested for deviation from uniform distribution with the Rayleigh test. Circular statistics (using CircStat, [42]) were used to estimate PH(100), the mean instantaneous phase at 100 ms postsound (the time of highest, sound-induced enhancement of phosphene rate in experiment 1; see Figure 1, left panel). The number of trials showing a phase within ±10 degrees of PH(100) was counted at 19 equally spaced time points in the window 30 ms to 300 ms, and the resulting time series was correlated with the mean phosphene detection rate from experiments 1 and 2.

For TMS-trials, EEG epochs between +20 ms (hence well beyond the brief TMS artifact, see [29]) and +500 ms after TMS stimulation, or between −500 and −20 ms preceding sound presentation (latter used for baseline correction), at each time delay after sound onset were band-pass filtered in the alpha range (8–14 Hz), then rectified. Alpha power values were then averaged across trials for each individual in each condition for a 100 ms critical time window extending from +100 to +200 ms after the TMS pulse [23], then baseline corrected to the corresponding cross-trial average in a presound interval. This procedure calculates event-related alpha changes and reflects visual cortex reactivity when computed for occipitoparietal sensors in response to visual input [22], or in response to the occipital TMS (see also [23]). For further details on EEG analysis, see Supplemental Information.

## Supplemental Information

Document S1. Figures S1 and S2, Supplemental Results and Discussion, and Supplemental Experimental Procedures
