# Supplementary material for: Sounds Reset Rhythms of Visual Cortex and Corresponding Human Visual Perception
Source: Curr Biol. 2012 May 8;22(9-2):807–13. doi: 10.1016/j.cub.2012.03.025 (PMC3368263; doi:10.1016/j.cub.2012.03.025)
Supplement: Document S1. Figures S1 and S2, Supplemental Results and Discussion, and Supplemental Experimental Procedures [file mmc1.pdf]

Current Biology, Volume *22*

Supplemental Information

Sounds Reset Rhythms of Visual Cortex

and Corresponding Human Visual Perception

Vincenzo Romei, Joachim Gross, and Gregor Thut

Supplemental Inventory

1. Supplemental Figures

Figure S1, related to Figure 2

Figure S2

2. Supplemental Results and Discussion

3. Supplemental Experimental Procedures

4. Supplemental References

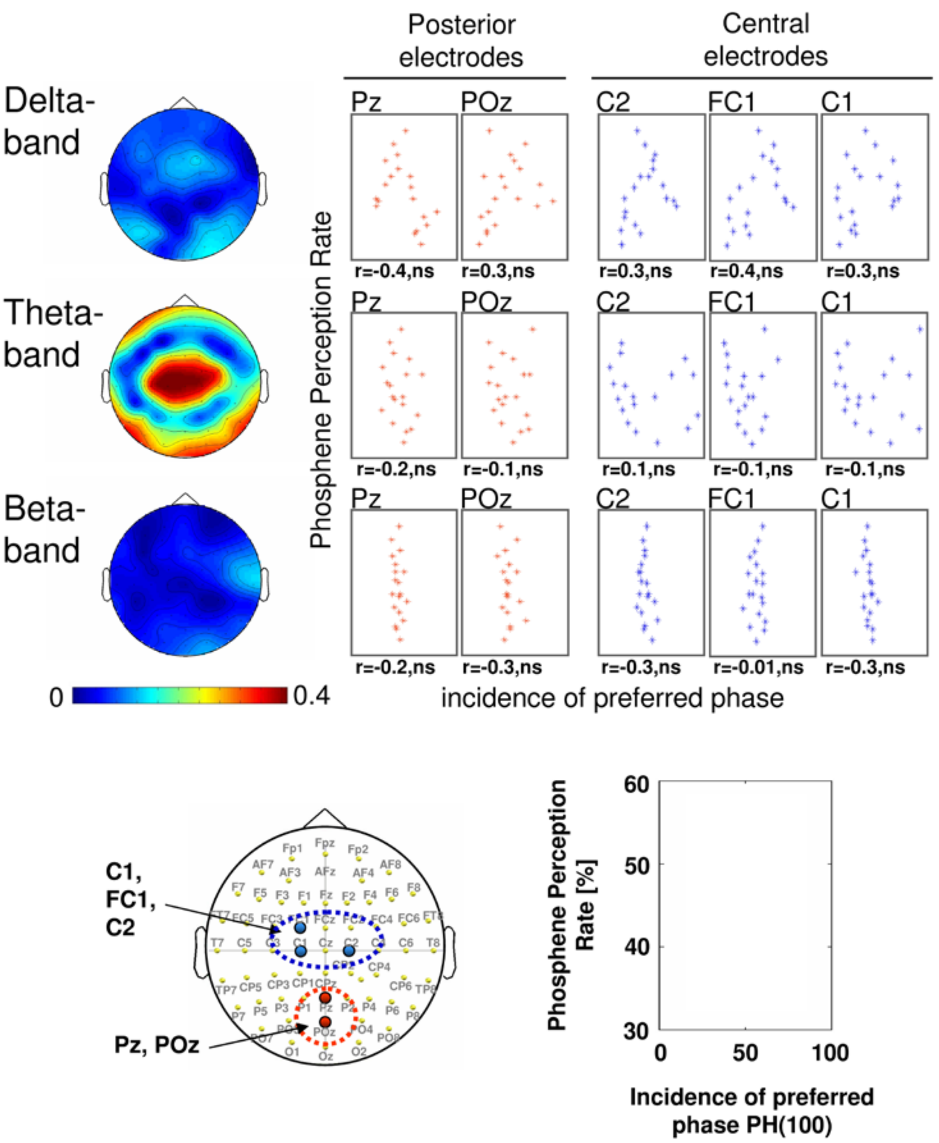

Figure S1. Sound Induced Phase-Locking in Nonalpha Frequency Bands, and Relation to Dynamics of Phosphene Perception, Related to Figure 2

Upper left: Topoplots show phase-locking values over the electrode array in the delta (2-4Hz), theta (4-6Hz) and beta frequency bands (17-23Hz) between 50-250ms after sound-onset (compare to alpha-topoplot in main Figure 2, upper right panel).

Upper right: Scatterplots of Phosphene perception rate in Exp 1 (y-axis) as a function of Incidence of preferred phase PH(100) in Exp. 2 (x-axis) per non-alpha frequency band. Scatterplots are shown for electrodes with posterior phase-locking maxima at alpha frequency (Pz and POz) (compare to scatterplot in main Figure 2, lower panel, red data points), as well as for electrodes with central phase-locking maxima at theta-frequency (C2, FC1, C1). Numbers below each scatterplot represent corresponding correlation coefficients (r) and p-values, all none significant.

Lower insets: Electrode positions, and scatterplot labels/scales.

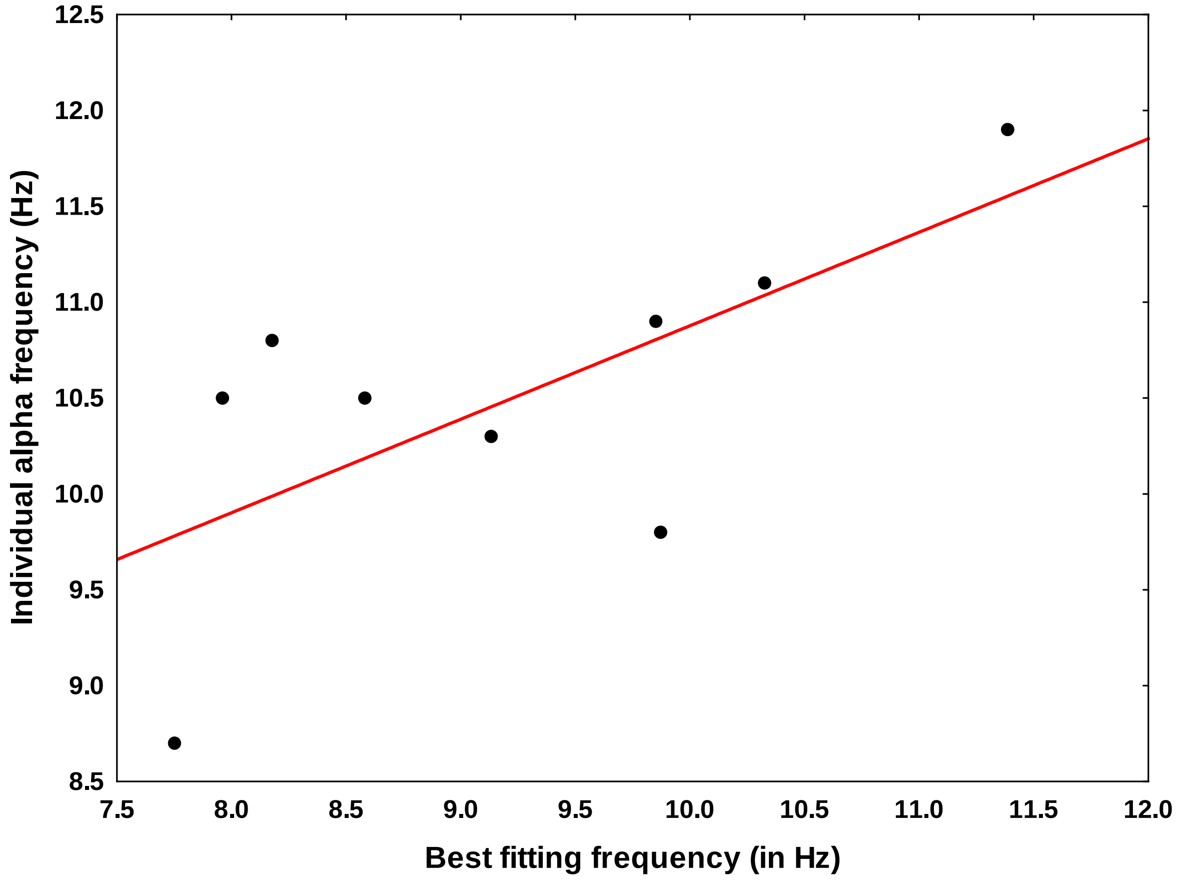

Figure S2. Periodicity in Phosphene Perception vs. Individual Alpha Frequency

Supplemental Results and Discussion

Control Analysis in Nonalpha (Flanker) Frequency Bands

To test whether the described relationship between sound-aligned oscillatory activity over posterior areas and phosphene perception rate is confined to the alpha-band (8-14Hz), or alternatively may span over other frequencies, we re-analyzed the EEG data of Experiment 2 using the same analysis stream (see Main text: EEG analysis in Experiment 2) but with bandpass filters set to isolate activity in adjacent delta- (2-4Hz), theta- (4-6Hz) and beta-frequency bands (17-23Hz) instead.

Analyzing other frequency bands helps to address the following three points. ***First***, it allows probing to what extent sound-aligned activity beyond the alpha-band may contribute to explain the cyclic pattern in phosphene perception (on top of the contribution from alpha-oscillations). Contribution from other frequency bands is conceivable as phase dynamics of slower frequencies have been linked to perception [3]. ***Second***, probing for effects beyond the alpha band allows ruling out a number of alternative explanations of the double peak in phosphene rate, which would not be consistent with sound-related alignment of alpha-oscillations, but rather with a superposition of two brain waves, simultaneously evoked by the sound but with distinct peaks. For instance, it has been suggested that early evoked potentials are composed of evoked alpha- but also theta- and beta-waves (see [43, 44]). It is therefore conceivable that the double peak in phosphene perception may reflect the superposition of a sound-aligned, early alpha-wave and a slower theta-wave, peaking at 90ms and 190ms respectively. Corroborating that effects are confined to the alpha-band would therefore rule out an explanation in terms of mixed-frequency evoked potentials, and instead support a link to recurrent peaks of an alpha-oscillation. ***Third***, corroborating specificity of the effects to the alpha-band would furthermore rule out spectral filter leakage, i.e. the possibility that some of the results may be carried by adjacent frequencies, rather than the alpha band.

The results of these additional analyses are illustrated in Supplemental Figure S1. The data reveal that sounds did phase-lock activity in other frequency bands. Most prominent non-alpha phase-locking occurred in the theta-band (4-6Hz) but this theta-phase locking showed a fronto-central maxima (Fig. S1, middle topoplot), i.e. was spatially dissociated from alpha-phase locking (suggesting locking of non-visual, possibly auditory activity). Phase-locking in the delta- and beta bands was much weaker (Fig. S1, upper and lower topoplots). Importantly, for none of these (non-alpha) frequency bands did we observe a significant correlation between the phase dynamics of PH100 in the 300ms epoch after sound-onset and the cyclic pattern of phosphene perception in the same window (see scatterplots in Fig S1, no consistent relationship across electrodes between incidence of preferred phase at100ms and phosphene rate across the 19 data point in this window, illustrated in Fig. S1 for posterior and central electrodes). Finally, we also looked at the incidence of preferred theta phase at 150ms (PH150), in order to explore whether a slow oscillation peaking/troughing at this delay may account for a general increase in phosphene perception. Again, we did not find a significant correlation between theta PH150 and behaviour in any electrode.

Taken together, we found no evidence for non-alpha (flanker) frequencies to contribute to the modulation of phosphene perception. The reported effects were therefore limited to the alpha frequency band. This rules out points 1-3 above, and further supports phase-locking of posterior alpha-oscillations as an explanation of the cyclic pattern of phosphene perception.

Correlation between Periodicity in Phosphene Perception and Individual Alpha-Frequency

To establish whether the periodicity of phosphene perception is related to the individual alpha frequency (IAF), we fitted a cosine function (y=a*cos(2*pi*b*x+c)+d) to each individual phosphene perception curve using non-linear least squares fitting in MATLAB. Explained variances ranged from 41%-77%, i.e. cyclic behavior was also present at the individual level. In parallel, we determined for each participant his/her individual alpha frequency using a Fast Fourier Transform (FFT) on artifact-free, 2-sec EEG-epochs prior to sound onset (using Brain Vision Software). We then correlated the resulting best fitting frequency (mean±SEM= 9.2±0.4Hz) with the obtained IAF (mean±SEM= 10.5±0.3Hz). This showed a significant positive correlation for both parametric (Pearson correlation: r=0.67, p=0.024, one-tailed) and non-parametric tests (Spearman rank: R=0.62, p=0.038, one tailed). Participants with low IAF showed slower cycling of phosphene rate and vice versa (see Fig. S2).

Supplemental Experimental Procedures

Participants

Fourteen healthy volunteers reporting normal hearing and normal or corrected vision were screened to identify those who reliably experience phosphenes induced by TMS over the occipital pole. Consistent with the literature [45], nearly 60% (9/16) did, so these 9 were selected to participate.

TMS Protocol

Using TMS over the occipital pole to induce illusory visual phosphene perceptions is now a well-established method that has been used extensively to examine excitability of human visual cortex [e.g. 10, 11, 14, 15, 28, 46 – 49], implicating early visual areas [e.g. 46, 49].

All participants underwent preliminary sessions in separate days prior to the experimental sessions, for careful determination of the dorsal occipital pole site at which a TMS pulse reliably induced a phosphene, and the minimum intensity needed to evoke this on ~50% of trials (i.e. Phosphene Threshold, PT). These sessions (all without the critical experimental sound) also served to confirm the consistency of phosphene perception over repeated trials, and of PT over sessions, which confirmed that the subsequent experimental session was feasible in the selected participants. The optimal TMS coil position over the occipital pole, as well as the shape, size and position of the perceived phosphenes, varied somewhat across participants, but was consistent for each across the different sessions. Phosphenes consistently appeared within the lower visual field quadrant, contralateral to stimulated occipital cortex, in correspondence with stimulation of the dorsal part of the occipital pole representing near-central parts of the lower visual field, as in previous reports [e.g. 10, 11, 14, 15, 28, 46 – 49].

An 85% sub-PT intensity was chosen for use in the two experiments on influences from the sound, as this intensity was previously shown to induce phosphene perception in ~50% of trials when single-pulse TMS is paired with brief auditory stimuli [10], indicating its suitability for preventing floor and ceiling effects here, as was successfully achieved. With mean PT across participants being 71.33% (±3.12) of maximum stimulator output (MSO) for the set-up in Experiment 1, the TMS intensity (85% PT) used during that experiment was 60.63% MSO (±2.65). Prior to Experiment 2, phosphene threshold (PT) was reassessed, since the electrode cap now came between the TMS stimulator and the skull. The mean PT across participants was now 73.56% MSO (±3.91). The corresponding stimulation intensity (85% PT) used during Experiment 2 was 63.56% MSO (±3.79).

Procedure

Sound and TMS delivery, plus behavioral response collection, were controlled by E-prime (E-Prime 1.1; Psychology Software Tools, Pittsburgh, PA). Participants pressed a button with right index finger to indicate phosphene perception, with right middle finger to indicate none. The intertrial interval varied pseudorandomly from 3000 to 5000 ms in steps of 500 ms, with each block lasting ~10 min. Breaks were encouraged between blocks. The blindfold was removed between TMS blocks in both experiments, to minimize systematic drifts in PT [50] by adaptation to darkness or drowsiness. For Experiment 1, 20 trials were assessed per participant per equiprobable condition, so that each participant completed 4 blocks of 100 trials, for a total of 400 trials. For Experiment 2, 96 trials were assessed per equiprobable condition, so that each participant completed 8 blocks of 96 trials for a total of 768 trials.

Phosphene Analyses

The timecourse of sound-induced changes in visual cortex excitability in Experiment 1 was expressed as percentage phosphene rate, then submitted to repeated measure Analysis of Variance (ANOVA), with relation to preceding sound as the within subject factor (TMS alone, or 30, 45, 60, 75, 90, 105, 120, 135, 150, 165, 180, 195, 210, 225, 240, 255, 270, 285, 300 ms delays since sound). Discrete windows for peaks and troughs were computed averaging across 4 consecutive interstimulus intervals as specified in main text. These windows were assessed via t-tests against baseline and Bonferroni corrected. An analogous analysis took place for the fewer timepoints in Experiment 2.

EEG Analyses

For EEG acquisition during TMS (Experiment 2), the design of the BrainAmp amplifier allows fine adaptation to TMS stimulus magnitude by adjusting its sensitivity and operational range. We used a sensitivity of 100 nV/bit (Signal range/resolution) and an Analog/Digital conversion range of 6553.5 uV (±3.277 mV), sufficient to prevent EEG signal saturation by the TMS Pulse [see 51]. Please note that the electric artifact due to a TMS pulse is very short-lived [c.f. 51] so cannot contribute to the timewindow used to assess TMS-related alpha-power changes in Experiment 2. This window was from +100 to +200 after each TMS pulse [see 23].

EEG recordings were processed using Fieldtrip [52], Circstat [42], Brain Vision (BrainProducts GmbH, Munich, Germany) and custom-made matlab functions. Data were visually inspected for artifacts and subsequently transformed to an average reference.

Supplemental References

43. Klimesch, W., Schack, B., Schabus, M., Doppelmayr, M., Gruber, W., and Sauseng, P. (2004). Phase-locked alpha and theta oscillations generate the P1-N1 complex and are related to memory performance. Brain Res. Cogn. Brain Res. 19, 302-316.

44. Gruber, W.R., Klimesch, W., Sauseng, P., and Doppelmayr, M. (2005). Alpha phase synchronization predicts P1 and N1 latency and amplitude size. Cereb. Cortex. 15, 371-377.

45. Kammer, T., Puls, K., Erb, M., and Grodd, W. (2005). Transcranial magnetic stimulation in the visual system. II. Characterization of induced phosphenes and scotomas. Exp. Brain Res. 160, 129-140.

46. Cowey, A., and Walsh, V. (2000). Magnetically induced phosphenes in sighted, blind and blindsighted observers. Neuroreport 11, 3269-3273.

47. Pascual-Leone, A., and Walsh, V. (2001). Fast backprojections from the motion to the primary visual area necessary for visual awareness. Science 292, 510-512.

48. Bestmann, S., Ruff, C.C., Blakemore, C., Driver, J., and Thilo, K.V. (2007). Spatial attention changes excitability of human visual cortex to direct stimulation. Curr. Biol. 17, 134-139.

49. Silvanto, J., Muggleton, N., Lavie, N., and Walsh, V. (2009). The perceptual and functional consequences of parietal top-down modulation on the visual cortex. Cereb. Cortex 19, 327-330.

50. Pitskel, N.B., Merabet, L.B., Ramos-Estebanez, C., Kauffman, T., and Pascual-Leone, A. (2007). Time-dependent changes in cortical excitability after prolonged visual deprivation. Neuroreport 18. 1703-1707.

51. Veniero, D., Bortoletto, M., and Miniussi, C. (2009). TMS-EEG co-registration: on TMS-induced artifact. Clin. Neurophysiol. 120, 1392-1399.

52. Oostenveld, R., Fries, P., Maris, E., and Schoffelen, J.M. (2011). FieldTrip: Open source software for advanced analysis of MEG, EEG, and invasive electrophysiological data. Comput. Intell. Neurosci. 2011, 156869.
